# Purification of Quinoline Insolubles in Heavy Coal Tar and Preparation of Meso-Carbon Microbeads by Catalytic Polycondensation

**DOI:** 10.3390/ma17010143

**Published:** 2023-12-27

**Authors:** Lei Zhang, Ruikang Song, Yang Jia, Zhuorui Zou, Ya Chen, Qi Wang

**Affiliations:** 1 School of Geology and Environment, Xi’an University of Science and Technology, Xi’an 710054, China; srk781388595@163.com (R.S.); 18209291062@163.com (Z.Z.); c1431332271@163.com (Y.C.); 18391871923@163.com (Q.W.); 2 Key Laboratory of Coal Resources Exploration and Comprehensive Utilization, Ministry of Natural Resources, Xi’an 710021, China; 3 State Key Laboratory of Eco-Hydraulics in Northwest Arid Region, Xi’an University of Technology, Xi’an 710048, China; 18329966966@sohu.com

**Keywords:** heavy coal tar, purification, catalytic polycondensation, quinoline insoluble, mesocarbon microbeads

## Abstract

The quinoline-insoluble (QI) matter in coal tar and coal tar pitch is an important factor affecting the properties of subsequent carbon materials. In this paper, catalytic polycondensation was used to remove QI from heavy coal tar, and meso-carbon microbeads could be formed during the purification process. The results showed that AlCl_3_ had superior catalytic performance to CuCl_2_, and the content of QI and heavy components, including pitch, in the coal tar was lower after AlCl_3_ catalytic polycondensation. Under the condition of catalytic polycondensation (AlCl_3_ 0.9 g, temperature 200 °C, and time 9 h), AlCl_3_ could reduce the QI content in heavy coal tar. The formed small particles could be filtered and removed, and good carbon materials could be obtained under the condition of catalytic polycondensation (AlCl_3_ 0.9 g, temperature 260 °C, and time 3 h).

## 1. Introduction

Coal is an important chemical raw material [1,2], and a great amount of by-product coal tar is produced from the coal chemical industry every year [3,4,5]. The production of low-temperature coal tar produced by the coal chemical industry is especially abundant in the northern region of Shaanxi, China [6,7]. Since the local coal is the raw material for producing high-quality semicoke, the tar content is high. There are tar-rich coals with yields greater than 7%, and even high-tar coals containing 12% [8,9]. Although the coal in northern Shaanxi is of good quality and low sulfur content, the prepared blue carbon is of good quality and has a wide market, but the classification and utilization of tar is still an important problem that local coal enterprises urgently need to solve [10,11,12].

Coal tar is distilled to extract different oil products and raw materials [13,14,15]. Although the heavy components can be fully utilized by means of catalysis or hydrogenation [16,17,18], coal tar pitch contains inorganic and organic quinoline-insoluble matter [19,20]. This makes the purification of coal tar pitch the key to further deep processing to prepare high-performance and high-value-added carbon materials [21,22].

The quinoline-insoluble particles in coal tar pitch are divided into primary quinoline insolubles and secondary quinoline insolubles [23]. The QI content is mainly composed of pyrolytic carbon, pitch coke, and tar solids, whereas the primary QI content is derived from the nanoparticles decomposed by hydrocarbons [24]. The difference in QI content will directly affect the performance of carbon materials and composite materials. And the existence of QI particles will reduce the viscosity of the system, which increases the difficulty of graphitizing subsequent carbon materials [25]. It is generally believed that the primary QI will limit the development of mesophases and affect the properties of carbon materials [26].

With the increasing requirements of carbon product quality in the market [27], the removal of QI particles has become particularly important, and the purification of coal tar pitch has important economic and strategic significance [28]. After removing heavy components (quinoline insolubles) and mixing aromatic and aliphatic solvents, a suitable starting material for the preparation of needle coke was obtained [29]. Through centrifugation and thermal filtration, purified coal tar was obtained [30]. Jung-Chul An et al. [31] used different paraffins to extract QI particles and found that high temperature and high pressure could improve the extraction reaction. The QI content of the purified coal tar pitch was low, and it could be prepared for needle coke. Ionic liquids were also beginning to be used as solvents for QI removal, and their extraction process was more environmentally friendly than that of conventional solvents [32]. However, the solvent used in the extraction process still caused solvent volatilization and environmental pollution [33,34,35].

It has been reported in the literature that the content of QI particles could also be adjusted by heat treatment [36]. As an important treatment method, it could convert the active components in the pitch into secondary quinoline insolubles, increasing the QI particle size and making it easier to remove [37]. Xingwei Zhang et al. [38] added naphthyl-based mesophase pitch to refined coal tar pitch, and the additive acted as a nucleating agent for thermal condensation to accelerate the reaction. Therefore, we used the Lewis acid catalyst AlCl_3_ to prepare mesocarbon microbeads (MCMBs), which were easier to filter and remove from the QI content of coal tar pitch by catalytic polycondensation. That method was in line with the existing two-carbon policy, and it could also provide reference ideas for the subsequent purification of coal tar pitch and the preparation of high-quality carbon materials.

## 2. Experimental

### 2.1. Materials

The tar used in the experiment was low-temperature coal tar (mainly underwater heavy coal tar), which was a by-product of semicoke produced in a coal chemical enterprise in northern Shaanxi. The coal used by the company was locally produced tar-rich coal, mainly from the Zhangjiamao, Ningtiaota, and Hongliulin mining areas. In order to ensure that the experimental process can accurately restore the real production environment, the temperature of the laboratory should be controlled at about 21–25 °C, the relative humidity should be controlled at 45–55%, and the tar should be stored in a polyethylene plastic barrel.

The supplier of AlCl_3_ is Damao Chemical Reagent Factory, Tianjin, China, and CuCl_2_ is provided by Shantou Xilong Chemical Industry Factory Co., Ltd., Guangdong, China.

### 2.2. Methods

#### 2.2.1. Purification and Separation of Quinoline Insolubles and Mesocarbon Microbeads from Heavy Coal Tar 

Using AlCl_3_ as a catalyst, a catalytic polycondensation method was used to cause heterogeneous nucleation of quinoline-insoluble (QI) particles in heavy coal tar, promoting the formation of mesophase small spheres to realize the purification of heavy coal tar. QI content in the initial heavy coal tar was measured, and then QI content after the purification was also measured. The purified heavy coal tar was separated from the meso-carbon microbeads by vacuum filtration. They were collected and weighed, and their yield was calculated. After measurement, the QI content of the original heavy coal tar was 0.1303 g, accounting for 1.303% of the total heavy coal tar.

#### 2.2.2. Composition Analysis of Heavy Coal Tar

To detect the coal tar, a simulated distillation method was adopted to analyze the distribution of each fraction, and it was carried out on a gas chromatograph. The instrument is produced by Beijing Purse General Instrument Co., Ltd. (GC1100) (Beijing, China). The working conditions included 99.999% nitrogen as the carrier gas, with a flow rate of 1 mL/min. The inlet temperature was 300 °C for 2 min, and the column oven temperature was 40 °C, which was gradually increased to 330 °C at 5 °C·min^−1^ and maintained for 3 min.

### 2.3. Orthogonal Experiment Design

An orthogonal experiment with three factors and four levels was designed, as shown in Table 1, after analysis, and the experiment was carried out according to the L16 (43) orthogonal array shown in Table 2. The three factors of AlCl_3_ addition (0 g, 0.3 g, 0.6 g, 0.9 g), heat polycondensation temperature (170 °C, 200 °C, 230 °C, 260 °C), and heat polycondensation time (3 h, 6 h, 9 h, 12 h) were investigated for their content of quinoline-insoluble (QI) particles. Additionally, the yield of MCMBs was also obtained from the factors and levels listed in the orthogonal table.

### 2.4. Characterization

The pulverized coal used in the test weighed 10 g. The working conditions for the determination of X-ray diffraction were a voltage of 36 kV and a current of 20 mA. The starting angle was 5°, and the ending angle was 120°. The step width was 0.02°; the wavelength was 1.788972 Å; and Cu was the target.

The Fourier infrared spectrometer was produced by Bruker (Billerica, MA, USA), and the model was VERTEX 70. The pulverized coal used in the test weighed 5 g. The test conditions were as follows: the scanning range of the infrared spectrometer was 4000–400 cm^−1^, the number of scans was 28, and the resolution was 0.4 cm^−1^.

The morphology of MCMB was observed using an electron microscope, model JSM-6460LV (produced by Japan Electron Beam Corporation (Tokyo, Japan)), in which the working voltage was 20 kV and the magnification was approximately 5000 times and 10,000 times.

## 3. Result and Discussion

### 3.1. Purification of QI Particles in Heavy Coal Tar by Catalytic Polycondensation

**Figure 1:**
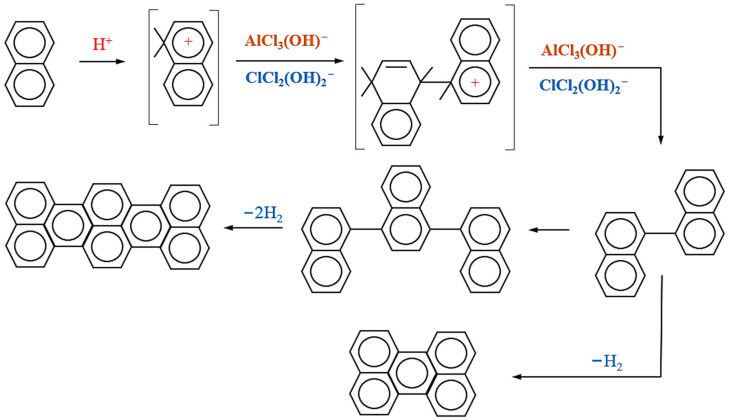
Mechanism of AlCl_3_ and CuCl_2_ catalytic polycondensation.

**Figure 2:**
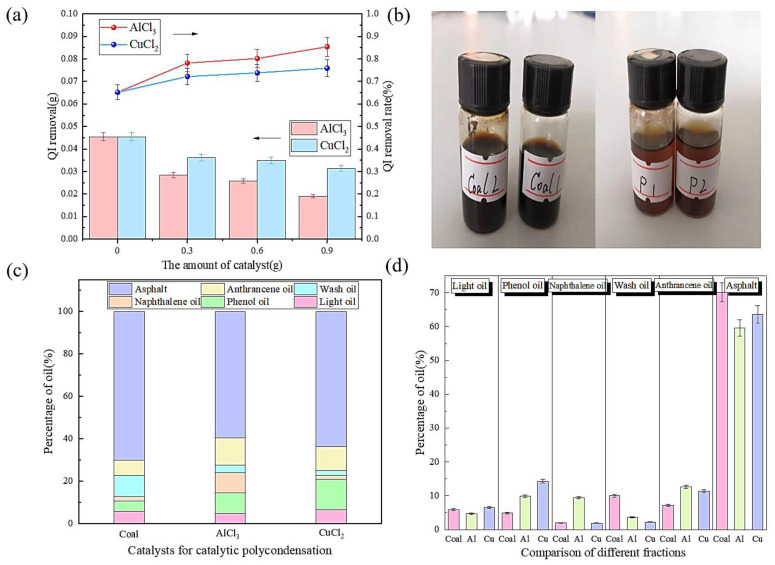
The effect of catalytic polycondensation on the purification effect of heavy coal tar: (**a**) is the relationship between QI content and QI removal rate; (**b**) is the comparison of heavy coal tar before and after purification under AlCl_3_ and CuCl_2_; (**c**) is the content of different fractions of heavy coal tar after purification; and (**d**) is the comparison of the content of different fractions of purified heavy coal tar.

Under conditions of 200 °C and 6 h, different amounts of catalysts were selected to catalyze the polycondensation of heavy coal tar. The relationship between the QI content of purified heavy coal tar and the amount of catalyst was explored, and the best catalyst was selected as the basis for subsequent research. In addition to inorganic substances in QI matter, there were also a large number of fused-ring aromatic structures. After adding the Lewis acid catalysts AlCl_3_ and CuCl_2_, a catalytic polycondensation reaction occurred. In the reaction process, part of the aliphatic side chain was removed, more alkyl groups and cycloalkyl groups were obtained, and a highly condensed, fused-ring macromolecular compound was formed. This catalytic polycondensation process is illustrated in Figure 1. It can be seen from Figure 2a that the QI content decreased after adding AlCl_3_ and CuCl_2_, and the use of an AlCl_3_ catalyst had a better QI removal effect. The comparison of heavy coal tar before and after purification is shown in Figure 2b. From its appearance, the color of unpurified raw, heavy coal tar was deep, while the color of the heavy coal tar after purification was lighter. The content of light fractions in purified heavy coal tar increased overall, as shown in Figure 2c. After purification with AlCl_3_, the content of phenol oil, naphthalene oil, and anthracene oil increased, while the content of light oil, washing oil, and pitch decreased. And after purification with CuCl_2_, the content of light oil, phenol oil, and anthracene oil increased, and the content of naphthalene oil, washing oil, and pitch decreased. This shows that in the purification of heavy coal tar, the Lewis acid catalysts AlCl_3_ and CuCl_2_ can reduce the activation energy of aromatic molecules and change the aromaticity of heavy coal tar [39,40]. Especially under the catalysis of AlCl_3_, the content of the pitch component in the heavy coal tar system decreased, as shown in Figure 2d.

According to the influence of polycondensation temperature, polycondensation time, and AlCl_3_ addition on the change in QI content, this paper established an orthogonal experiment table of 3 factors and 4 levels. The experiment was carried out according to the conditions in Table 3, and the measured QI content was used as the experimental result.

The range analysis of QI content in Table 3 was obtained through calculation. K_j_ is the sum of each factor at the same level, and k_j_ is the mean value at the same level; that is, k_j_ = K_j_/4. Through the range analysis, the range (R) value was obtained by Equation (1). The value of R represents the importance of the factors, so the importance of the three factors in descending order is time, AlCl_3_ addition, and temperature. According to the experimental results of the QI content, the result of experiment number 14 was the best. And the corresponding factor was the best experimental condition factor; that is, when the AlCl_3_ addition was 0.9 g, the temperature was 230 °C, and the time was 9 h. After the catalytic polycondensation of heavy coal tar, the QI content was the lowest.

(1) $$R=\max\left(k_{j}\right)-\min\left(k_{j}\right)\;(j={\mathrm{AlCl}}_{3}\;\mathrm{addition},\;\mathrm{Temperature},\;\mathrm{Time};\;\mathrm{and}\;j=1,\;2,\;3,\;4)$$

In addition, the variance analysis of each factor was also analyzed for the QI content. In Table 4, SS is the sum of squared deviations of various levels under different factors; MS is mean square; Df is degrees of freedom; and the F value is the ratio of MS in different factors to that of the error column. The *p*-value is the possibility that it may be greater than the F value. In general, when the *p*-value was less than 0.05, it was considered that the factor had a significant impact on the experimental results and it was an important indicator affecting the experimental indicators. According to the calculation results, it is known that the AlCl_3_ addition, temperature, and time all had a significant effect on the removal of QI by the catalytic polycondensation of heavy coal tar. And all parameters were calculated using relevant functions in Excel.

**Figure 3:**
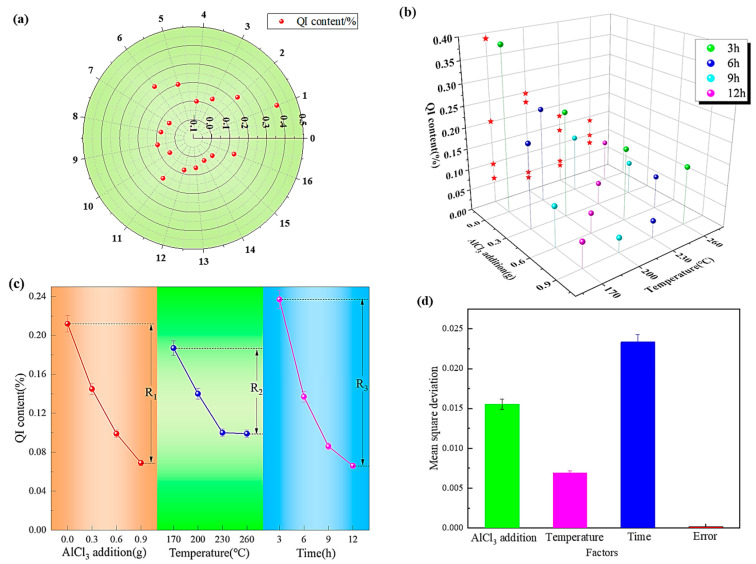
The effect of different conditions on the removal of QI by catalytic polycondensation: (**a**) is the distribution of QI content under different experiment numbers; (**b**) is projection of QI content on YZ axis under different conditions; (**c**) is the trend of QI content under different conditions; and (**d**) is mean square deviation of factors at different levels.

The relationship between different experiment numbers and QI content is shown in Figure 3a. The smaller the QI content, the better the heavy coal tar purification effect, and this shows more as it gets closer to the center of the circle in the figure. As also shown in Figure 3b, the projection of the minimum QI content of three different factors on the YZ axis can illustrate the combination of the optimal level and the optimal scheme. Figure 3c illustrates the effect of QI content under different factors, and it illustrates the influence and trend of thermal polycondensation conditions on QI content under different factors. And the difference between the maximum and minimum QI content is precisely the R value in the orthogonal experiment table. The effects of each factor on average-level mean square deviation are shown in Figure 3d, in which the abscissa is related to the four factors corresponding to AlCl_3_ addition: temperature, time, and error, successively.

### 3.2. Preparation of Mesophase Carbon Microspheres by Catalytic Polycondensation

**Figure 4:**
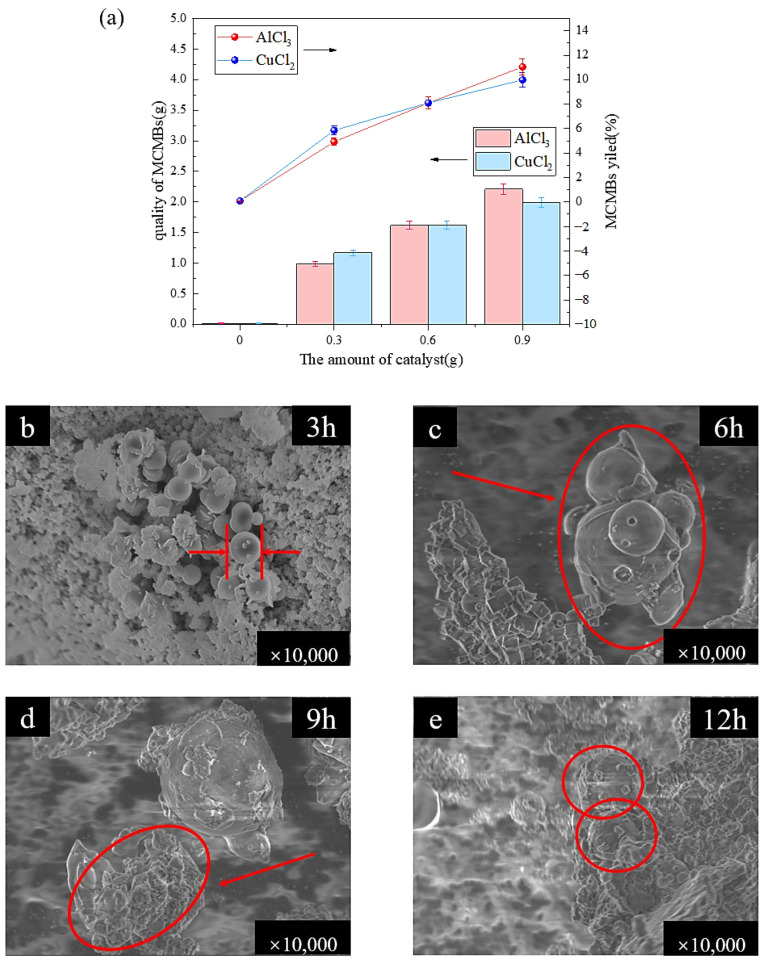
The effect of catalytic polycondensation to prepare MCMBs of heavy coal tar: (**a**) is the relationship between the quality of MCMBs and MCMB yields; (**b**) is the morphology of spheres after 3 h of catalytic polycondensation; (**c**) is the morphology of spheres after 6 h of catalytic polycondensation; (**d**) is the morphology of spheres after 9 h of catalytic polycondensation; and (**e**) is the morphology of spheres after 12 h of catalytic polycondensation.

Under the conditions of 200 °C and 6 h, different amounts of catalysts were selected to catalyze the polycondensation of heavy coal tar to prepare the MCMBs. The different Lewis acids, AlCl_3_ and CuCl_2_, had a significant effect on the MCMB yields. It can be seen from Figure 4a that after adding the Lewis acid catalyst, the MCMB yields were significantly improved. When the addition amount of catalyst was 0.9 g, the MCMB yields were the highest, which were 11.06% and 9.98%, respectively.

Samples were taken at different polycondensation times to observe the morphology of the MCMBs. The growth process of the mesosphere spheres at different temperatures is shown in Figure 4b–e. The added AlCl_3_ would act as a primary QI, which had a positive effect on the formation of the MCMBs, and some related articles had been reported. That was the main reason that the added inorganic matter caused heterogeneous nucleation of tar during polycondensation, and the inorganic matter acted as the nucleating agent [41]. It reduced the surface energy of the heavy coal tar system and adhered to the surface of the spheres to prevent the growth and fusion between spheres [42], which was also the reason why the surface of the small spheres was not smooth, as shown in Figure 4c.

Similar to the QI content in the catalytic polycondensation to purify heavy coal tar, it established an orthogonal experiment table of 3 factors and 4 levels (polycondensation temperature, polycondensation time, and AlCl_3_ addition) on the MCMB yield. And Table 5 shows the factors affecting the MCMB yield and its results. The R-value showed that the AlCl_3_ addition, temperature, and time all had a significant effect on the formation of MCMBs by the catalytic polycondensation of heavy coal tar.

In Table 6, the *p* value of the catalytic polycondensation time is greater than 0.05, indicating that the effect of time on the preparation of MCMB is not obvious when the heavy coal tar catalytic polycondensation is used to prepare MCBM, and it mainly lies in the catalyst and temperature, which is consistent with previous reports.

**Figure 5:**
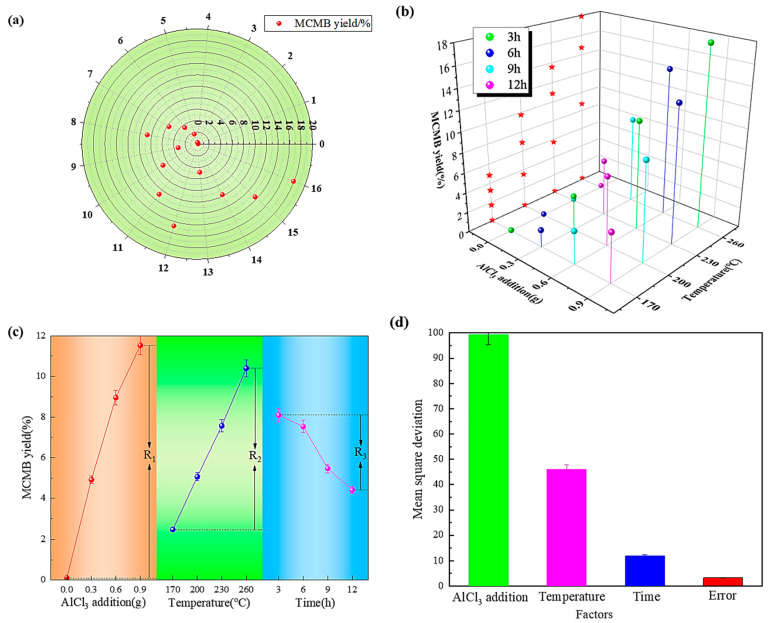
The effect of different conditions on the MCMB yields by catalytic polycondensation: (**a**) is the distribution of MCMB yields under different experiment numbers; (**b**) is projection of MCMB yields on YZ axis under different conditions; (**c**) is the trend MCMB yields under different conditions; and (**d**) is mean square deviation of factors at different levels.

The distribution of MCMB yield under different experiment numbers is shown in Figure 5a. Under the optimal catalytic polycondensation conditions (catalyst 0.9 g, catalytic polycondensation temperature 200 °C, catalytic polycondensation time 9 h), the content of QI was the lowest, which was 0.035%. Under these optimal conditions, the MCMB yield was only 9.76%, and it did not reach the maximum value of 17.91%. As also shown in Figure 5b, the projection of the maximum MCMB yield of three different factors on the YZ axis can illustrate the combination of the optimal level and the optimal scheme. Figure 5c illustrates the effect of MCMB yield under different factors, and it illustrates the influence and trend of thermal polycondensation conditions on MCMB yield under different factors. The difference between the maximum and minimum MCMB yield is precisely the R value in the orthogonal experiment table. The effects of each factor on average-level mean square deviation are shown in Figure 5d, in which the abscissa is related to the four factors corresponding to AlCl_3_ addition, temperature, time, and error, successively.

## 4. Conclusions

Using catalytic polycondensation by the Lewis acid catalysts AlCl_3_ and CuCl_2_, it was found that AlCl_3_ was more effective in purifying QI and could reduce the pitch content of coal tar. Under the condition of catalytic polycondensation (AlCl_3_ 0.9 g, temperature 200 °C, and time 9 h), the content of QI was reduced to 0.035%. And according to the orthogonal experimental analysis, the polycondensation time had more influence on the removal of QI by catalytic polycondensation. Under the condition of catalytic polycondensation (AlCl_3_ 0.9 g, temperature 260 °C, and time 3 h), the content of MCMBs was the highest, up to 17.91%. The amount of catalyst showed more obvious effects, according to the orthogonal experimental analysis.

## Figures and Tables

**Table 1:** Orthogonal factor and level table.

Level	Factors		
	AlCl_3_ Addition (g)	Temperature (°C)	Time (h)
1	0	170	3
2	0.3	200	6
3	0.6	230	9
4	0.9	260	12

**Table 2:** L_16_(4^3^) Orthogonal array.

Level	Factors		
	AlCl_3_ Addition (g)	Temperature (°C)	Time (h)
1	1	1	1
2	1	2	2
3	1	3	3
4	1	4	4
5	2	1	2
6	2	2	1
7	2	3	4
8	2	4	3
9	3	1	3
10	3	2	4
11	3	3	1
12	3	4	2
13	4	1	4
14	4	2	3
15	4	3	2
16	4	4	1

**Table 3:** Orthogonal designed table of QI content and its results.

Experiment Number	Factors				
	AlCl_3_ Addition (g)	Temperature (°C)	Time (h)	Error	QI Content (%)
1	1 (0)	1 (170)	1 (3)	1	0.388
2	1	2 (200)	2 (6)	2	0.227
3	1	3 (230)	3 (9)	3	0.135
4	1	4 (260)	4 (12)	4	0.099
5	2 (0.3)	1	2	4	0.201
6	2	2	1	3	0.248
7	2	3	4	2	0.053
8	2	4	3	1	0.078
9	3 (0.6)	1	3	2	0.097
10	3	2	4	1	0.049
11	3	3	1	4	0.172
12	3	4	2	3	0.079
13	4 (0.9)	1	4	3	0.061
14	4	2	3	4	0.035
15	4	3	2	1	0.041
16	4	4	1	2	0.139
K1	0.849	0.747	0.947	0.555	
K2	0.580	0.559	0.548	0.538	
K3	0.397	0.401	0.345	0.524	
K4	0.276	0.395	0.262	0.485	
k1	0.212	0.187	0.237	0.139	
k2	0.145	0.140	0.137	0.135	
k3	0.099	0.100	0.086	0.131	
k4	0.069	0.099	0.066	0.121	
R	0.143	0.088	0.171	0.018	

**Table 4:** Variance analysis of effect of polycondensation conditions on QI content.

Evaluation Indexes	Factors			
	AlCl_3_ Addition (g)	Temperature (°C)	Time (h)	Error
SS	0.0466	0.0207	0.0700	0.0010
Df	3	3	3	6
MS	0.0155	0.0069	0.0233	0.0002
F-value	96.7229	43.0197	145.3975	/
*p*-value	0.00002	0.00019	0.00001	/

**Table 5:** Orthogonally designed table of MCMB yield and its results.

Experiment Number	Factors				
	AlCl_3_ Addition (g)	Temperature (°C)	Time (h)	Error	MCMB Yield (%)
1	1 (0)	1 (170)	1 (3)	1	0.059
2	1	2 (200)	2 (6)	2	0.087
3	1	3 (230)	3 (9)	3	0.121
4	1	4 (260)	4 (12)	4	0.179
5	2 (0.3)	1	2	4	1.71
6	2	2	1	3	3.51
7	2	3	4	2	5.69
8	2	4	3	1	8.75
9	3 (0.6)	1	3	2	3.28
10	3	2	4	1	6.91
11	3	3	1	4	10.88
12	3	4	2	3	14.75
13	4 (0.9)	1	4	3	4.85
14	4	2	3	4	9.76
15	4	3	2	1	13.58
16	4	4	1	2	17.91
K1	0.446	9.899	32.359	30.259	
K2	19.660	20.267	30.127	24.567	
K3	35.820	30.271	21.911	26.651	
K4	46.100	41.589	17.629	20.549	
k1	0.112	2.475	8.090	7.565	
k2	4.915	5.067	7.532	6.142	
k3	8.955	7.568	5.478	6.663	
k4	11.525	10.397	4.407	5.137	
R	11.414	7.923	3.683	2.428	

**Table 6:** Variance analysis of effect of polycondensation conditions on MCMB yield.

Evaluation Indexes	Factors			
	AlCl_3_ Addition (g)	Temperature (°C)	Time (h)	Error
SS	298.168	138.098	35.822	19.979
Df	3	3	3	6
MS	99.3892	46.0328	11.9407	3.3298
F-value	29.8484	13.8245	3.5860	/
*p*-value	0.0083	0.0255	0.1605	/

## Data Availability

The data presented in this study are available on request from the corresponding author.
